# A case series of 37 surgically managed, paraplegic, deep pain negative French bulldogs, with thoracolumbar intervertebral disc extrusion, from two English referral centres

**DOI:** 10.1002/vro2.61

**Published:** 2023-05-09

**Authors:** Gareth Michael Couper Jones, Giunio Bruto Cherubini, Francisco Llabres‐Diaz, Abby Caine, Alberta De Stefani

**Affiliations:** ^1^ Department of Clinical Science and Services Royal Veterinary College Hertfordshire UK; ^2^ Dick White Referrals Station Farm Six Mile Bottom Cambridgeshire UK; ^3^ Veterinary Teaching Hospital “Mario Modenato” Department of Veterinary Sciences University of Pisa Pisa Italy

## Abstract

**Background:**

Thoracolumbar intervertebral disc extrusions (TL‐IVDEs) are a common spinal disorder in dogs, especially within chondrodystrophic breeds. Loss of deep pain perception is a well‐documented negative prognostic indicator in dogs with TL‐IVDE. The objectives of this study were to report the rate of return of deep pain perception and independent ambulation in surgically treated, paraplegic, deep pain perception negative French bulldogs with TL‐IVDEs.

**Methods:**

A retrospective case series of deep pain perception negative dogs with TL‐IVDE presenting to two referral centres between 2015 and 2020 was conducted. Medical and MRI records were reviewed, including the following quantitative MRI changes: lesion length, extent of spinal cord swelling and severity of spinal cord compression.

**Results:**

Thirty‐seven French bulldogs met the inclusion criteria, with 14 of 37 (38%) regaining deep pain perception by the time of discharge (median hospitalisation 10.0 days [interquartile range 7.0–15.5 days]) with two dogs independently ambulatory (6%). Ten of the 37 dogs were euthanased during hospitalisation. Significantly fewer dogs (3/16, 19%) with L4‐S3 lesions regained deep pain perception compared to 11 of 21 (52%) of dogs with T3‐L3 lesions (*p* = 0.048). Quantitative MRI changes were not associated with the return of deep pain perception. After discharge, with a median 1‐month follow‐up period, an additional three dogs regained deep pain perception and five dogs became independently ambulatory (17/37 [46%] and 7/37 [19%], respectively).

**Conclusions and clinical importance:**

This study adds support to the contention that the recovery of French bulldogs with TL‐IVDE from surgery is poor compared with other breeds; further prospective, breed‐controlled studies are indicated.

## INTRODUCTION

Acute thoracolumbar intervertebral disc extrusions (TL‐IVDEs), otherwise referred to as Hansen type I extrusions,^1^ are the most common neurological condition leading to acute neurological signs in dogs.^2^, ^3^ Thoracolumbar intervertebral disc extrusions can cause a spectrum of neurological impairments, ranging from spinal hyperaesthesia to paraplegia with loss of deep pain perception (DPP).^1^, ^3^ While TL‐IVDEs can occur in all breeds of dogs, they are more common in chondrodystrophic breeds, including Dachshunds, Cocker Spaniels and French bulldogs,^1^, ^4^, ^5^ with chondroid metaplasia of the intervertebral disc implicated.^4^

The presence of DPP has been identified as a key clinical prognostic indicator in dogs presenting with TL‐IVDE, with the rate of recovery following surgical management reported to be in excess of 90%.^6^ When DPP is absent, the rate of recovery following surgical management, defined as independent walking, ranges from 30% to 75% across several studies with varying long‐term follow‐up,^7^, ^8^, ^9^, ^10^, ^11^, ^12^ making the presence of DPP a key clinical prognostic indicator. However, most of these studies have an overwhelming prevalence of Dachshunds within their study populations, with limited information on French bulldogs. One report highlighted a potentially worse neurological outcome in French bulldogs compared with Dachshunds, with only 33% of French bulldogs without DPP regaining independent ambulation compared to 53% of Dachshunds. Only 15 French bulldogs, however, were included in that study.^8^ That study also identified that French bulldogs with TL‐IVDE presented at a younger age have a higher prevalence of myelomalacia and a higher frequency of L4‐S3 TL‐IVDE compared to Dachshunds.^8^ Other studies have also reported that French bulldogs have a high rate of late‐onset recurrence of clinical signs after apparently successful surgical management^13^ and that they have a higher incidence of extensive epidural haemorrhage compared to Dachshunds.^14^ This highlights that, as a breed, their clinical manifestation of TL‐IVDE disease may be different compared to other chondrodystrophic breeds.

Several other prognostic indicators have been investigated for dogs with TL‐IVDE ranging from clinical presentation, blood and cerebrospinal fluid biomarkers, to diagnostic imaging changes.^6^ Diagnostic imaging changes/biomarkers have been extensively investigated, with a particular focus on prognostic factors related to improvement in severely affected animals (such as paraplegic dogs) or animals with suspected progressive myelomalacia.^11^, ^15^, ^16^, ^17^, ^18^, ^19^, ^20^, ^21^, ^22^, ^23^, ^24^ Some authors have suggested that the presence and length of a T2‐weighted (T2W) signal increase in the spinal cord parenchyma is associated with a poorer clinical outcome,^21^, ^24^ although this is contested by others.^23^ Others have investigated both the severity and length of compression,^10^, ^17^, ^21^ with one report identifying an association between the compression length and neurological grade at presentation, although no association was found between this imaging marker and return of ambulation.^21^ These parameters, with the addition of length of spinal cord swelling, have also been investigated in cases with progressive myelomalacia with varying results.^15^, ^19^ Despite the lack of generalised consensus about the prognostic value of these measurements, with sometimes conflicting results in different studies, these MRI biomarkers are measured relatively easily based on routinely obtained sequences, independently of magnet strength. With further studies evaluating the use of imaging markers for disease classification and potential prognosis correlation, their potential benefit can be further interrogated if the most frequently evaluated markers are consistently measured.

Clinically, there is a general perception that French bulldogs have a poorer rate of recovery compared to Dachshunds and other chondrodystrophic breeds; however, the rate of recovery of DPP‐negative French bulldogs with TL‐IVDE has not been comprehensively explored. Given the associated morbidity and mortality of dogs with severe TL‐IVDE, as well as the large financial and time commitments from their caregivers, investigation into the specific rate of recovery for French bulldogs is warranted to aid clinical decision making. As such, the purpose of this study was to report the rates of return for DPP and independent ambulation in paraplegic, DPP‐negative French bulldogs that underwent surgical management.

## MATERIALS AND METHODS

### Cases

Medical records of dogs that were presented to the authors’ clinics for TL‐IVDEs between 2015 and 2020 were identified and reviewed. Three inclusion criteria had to be met: (1) being a French bulldog with a confirmed diagnosis of TL‐IVDE; (2) a neurological examination identifying paraplegia with absence of DPP; (3) undergoing surgical management. Dogs were excluded if their medical records were incomplete. For inclusion in the MRI imaging analysis, dogs were required to have retrievable MRI examinations, including at least T2W and short tau inversion recovery (STIR) sagittal images as well as T2W transverse images.

### Data collection

The following information was retrieved from the medical records and tabulated using electronic spreadsheet software (Microsoft Excel, Version 16.54, Microsoft, Washington, DC, USA): age, bodyweight, sex and neuter status, previous history of TL‐IVDE, neurological grade based on the modified Frankel score (MFS) at presentation,^9^, ^25^ results of diagnostic imaging findings, total general anaesthesia (GA) time, presence of complications, length of hospitalisation and final neurological grade at the time the dog was discharged or was euthanased/died. To investigate the interaction of these co‐variates, cases were separated into two groups based on whether they had regained DPP (a successful outcome) or not regained perception (unsuccessful outcome) at the time of discharge or the last neurological examination prior to death.

Independent ambulation was not used to categorise cases due to the small number of dogs able to ambulate at discharge. The clinical management of the case was at the attending veterinary clinician's discretion, including the duration of hospitalisation. Where available, longer term follow‐up data were collected.

### Quantitative MRI analysis

Digital imaging and communications in medicine (DICOM) images of the dogs that had fulfilled the inclusion criteria were downloaded and contemporaneously evaluated by two veterinary radiologists blinded to the clinical status of the dogs at the time of image acquisition and clinical outcome. The images were evaluated using Osirix (Version 12.5, Pixmeo SARL, Geneva, Switzerland). The MRI images were obtained using either a Philips Achieva 1.5T scanner (Philips, Guilford, UK) or a Hitachi Aperto 0.4T scanner (Aperto Lucent, Hitachi Medical Corporation, Tokyo, Japan). In addition to the sequences listed as inclusion criteria, high‐field images included the use of a 3D balanced turbo gradient echo sequence in most cases, generating thin slices with high contrast resolution. These images were used, if available. Sequence parameters, including slice thickness, as well as the final selection of coil and sequences, including the use of contrast, were influenced by the dimensions of the dog and personal preferences of the attending neuro‐radiology team. Complete evaluation of all obtained sequences was not performed for the purpose of this study.

The criteria for MRI analysis were evaluated for each case, and a consensus was reached for the criteria used in the analyses (see Table 1). For further information regarding the MRI criteria, including exemplar figures, see Figures S1–S5.

**TABLE 1:** Description of quantitative MRI biomarkers for thoracolumbar intervertebral disc disease used in the study.

Feature	Definition
T2W signal increase length/L2	Length of intramedullary T2W signal increase as measured on the sagittal view, standardised to the length of the body of L2
Extradural compression length /L2	Length of extradural compression as measured on the sagittal view, standardised to the length of the body of L2
Cord swelling length/L2	Length of spinal cord swelling as measured on the sagittal view, standardised to the length of the body of L2
Percentage of compression	Severity of compression, by comparing the height or width of the spinal cord at the site of maximal compression, to the same measurement of the ‘normal’ spinal cord at the centre of the vertebral body cranial to the last compressive lesion

### Statistical analysis

Data analysis was performed using SPSS (Version 27, IBM, New York, NY, USA). Categorical variables were described using frequencies and statistical analysis was performed using Fisher's exact test. For continuous variables, normality was tested using the Shapiro–Wilks test, and statistical analysis was performed using an unpaired Student's *t*‐test with or without Welch correction or a Mann–Whitney *U*‐test. Statistical significance was set as *p*‐value 0.05 or less.

## RESULTS

### Study population

Thirty‐seven (*n* = 37) French bulldogs met the inclusion criteria, with a median age of 3.9 years (interquartile range [IQR] 3.0–4.9 years) and a mean bodyweight of 12.8 kg (SD ±1.9 kg) at presentation. Male dogs accounted for 18 of 37 dogs in the study, with five of 18 being entire. Of the 19 female dogs in the study, seven were entire. Lesions within the T3‐L3 spinal segment accounted for 21 of 37 (57%) of the included cases, with 16 of 37 (43%) included cases having lesions within the L4‐S3 spinal segments.

Twenty‐seven dogs (27/37, 73%) were discharged alive from hospital. The remaining 10 dogs (27%) were euthanased during the post‐operative period, with four dogs euthanased due to suspected progressive myelomalacia, one dog euthanased due to severe respiratory dysfunction following aspiration pneumonia and the remaining five dogs euthanased at the primary care givers’ request due to lack of improvement.

The median duration of hospitalisation for the study population was 10.0 days (IQR 7.0–15.5 days). The duration of hospitalisation for each dog is reported in Table 2.

**TABLE 2:** Individual outcome data including spinal segment involved, length of hospitalisation, time taken for return of deep pain perception (DPP) and follow‐up data when available.

Case number	Spinal segment	Hospitalisation (days)	Grade at discharge (MFS)	Time for return of DPP (days)	Alive at discharge	Follow‐up available	Follow‐up time (months)	Neurological grade at follow‐up (MFS)	Reason for euthanasia (if applicable)
1	T3‐L3	18	3	<8	Yes	Yes	1	3	n/a
2	T3‐L3	21	5	n/a	No	n/a	n/a	n/a	No neurological improvement
3	T3‐L3	11	5	n/a	Yes	Yes	1	5	n/a
4	L4‐S3	5	5	n/a	No	n/a	n/a	n/a	No neurological improvement
5	T3‐L3	18	3	5	Yes	Yes	1	2	n/a
6	L4‐S3	3	5	n/a	No	n/a	n/a	n/a	Suspected progressive myelomalacia
7	T3‐L3	15	5	n/a	No	n/a	n/a	n/a	No neurological improvement
8	L4‐S3	10	3	2	Yes	Yes	1	2	n/a
9	L4‐S3	16	5	n/a	Yes	No	n/a	n/a	n/a
10	T3‐L3	15	5	n/a	Yes	Yes	1.5	5	n/a
11	T3‐L3	6	5	n/a	No	n/a	n/a	n/a	Suspected progressive myelomalacia
12	L4‐S3	5	5	n/a	Yes	Yes	>12	5	n/a
13	L4‐S3	16	5	n/a	No	n/a	n/a	n/a	No neurological improvement
14	T3‐L3	8	4	1	Yes	Yes	1	2	n/a
15	T3‐L3	10	5	n/a	Yes	Yes	3	4	n/a
16	L4‐S3	8	5	n/a	Yes	Yes	1	2	n/a
17	L4‐S3	16	5	n/a	Yes	Yes	2	5	n/a
18	T3‐L3	11	5	n/a	Yes	No	n/a	n/a	Euthanased at home, no neurological improvement
19	T3‐L3	15	3	5	Yes	Yes	>12	2	n/a
20	L4‐S3	4	5	n/a	No	n/a	n/a	n/a	Severe respiratory dysfunction
21	L4‐S3	5	5	n/a	Yes	Yes	3	5	n/a
22	L4‐S3	7	5	n/a	Yes	Yes	2	4	n/a
23	T3‐L3	13	5	n/a	Yes	Yes	2	5	n/a
24	L4‐S3	27	5	n/a	Yes	No	n/a	n/a	Euthanased at home, suspected progressive myelomalacia
25	T3‐L3	18	3	1	Yes	Yes	1	3	n/a
26	T3‐L3	17	2	2	Yes	Yes	1	2	n/a
27	T3‐L3	13	3	2	Yes	Yes	1	3	n/a
28	T3‐L3	3	5	n/a	No	n/a	n/a	n/a	Suspected progressive myelomalacia
29	L4‐S3	8	5	n/a	No	n/a	n/a	n/a	No neurological improvement
30	L4‐S3	10	4	1	Yes	Yes	1	3	n/a
31	T3‐L3	7	3	1	Yes	Yes	1	3	n/a
32	L4‐S3	6	3	1	Yes	Yes	1	3	n/a
33	T3‐L3	9	2	1	Yes	Yes	1	2	n/a
34	L4‐S3	10	5	n/a	No	n/a	n/a	n/a	Suspected progressive myelomalacia
35	T3‐L3	11	3	5	Yes	Yes	1	3	n/a
36	T3‐L3	12	5	n/a	Yes	Yes	3	5	n/a
37	T3‐L3	8	4	7	Yes	Yes	1	3	n/a

Abbreviation: MFS, modified Frankel score.

### Clinical outcome

Fourteen dogs (38%) had regained DPP (their last neurological examination MFS was less than 5), with two of 37 (6%) dogs able to independently ambulate at discharge.

Nearly half of the dogs (46%) that regained DPP (where data were available within the study period) did so within 1 day of surgery, with the median time for DPP to return following surgery being 2 days (IQR 1–5 days). The time taken for DPP to return in one dog was not clear from the clinical notes, although DPP had returned within 8 days of surgery. The time taken for DPP to return for 14 dogs is reported in Table 2.

The spinal segments involved were associated with the return of DPP. Significantly fewer dogs with L4‐S3 lesions regained DPP (3/16, 19%) compared to 11/21 (52%) dogs with T3‐L3 lesions (*p* = 0.048). Progressive myelomalacia was clinically suspected in five of 37 (14%) of dogs, with three of these dogs having L4‐S3 lesions. There was no association between regaining DPP and the following variables (*p* > 0.05): age, bodyweight and total GA time.

Limited follow‐up data were available for 24 discharged dogs, with a median follow‐up time of 1 month post‐surgery. The neurological grade as well as length of follow‐up are listed in Table 2. Three cases were lost to follow‐up, with two dogs discharged for home euthanasia and one dog not seen by their referring practice after discharge. Follow‐up data identified that an additional three dogs regained DPP, increasing the number of dogs who regained DPP to 17 (of 37; 46%). The number of dogs that could ambulate independently increased from two (of 37; 6%) to seven (of 37; 19%). In 13 discharged cases, there were no follow‐up data available about the time for return of DPP.

### Quantitative MRI analysis

For the quantitative MRI analysis, dogs were separated into two groups (successful [*n* = 14] and unsuccessful [*n* = 21]), with the results summarised in Table 3. Imaging data were not available for two dogs (both failed to regain DPP). There was no statistical significance for any quantitative MRI measurement between the two groups (*p* > 0.05).

**TABLE 3:** MRI measurements for French bulldogs that did and did not show deep pain perception (DPP) at their last neurological examination following surgical management for severe thoracolumbar intervertebral disc disease.

	Median (IQR)		
MRI parameter	‘Successful’ (regained DPP [*n* = 14])	‘Unsuccessful’ (did not regain DPP [*n* = 21])	*p*‐Value
T2W length/L2	6.28 (5.52–9.86)	7.64 (6.75–9.86)	0.127
	**Mean ± SD**	**Mean ± SD**	
Compression length/L2	4.01 ± 1.54	3.99 ± 1.66	0.955
Cord swelling length/L2	7.16 ± 2.38	8.38 ± 1.63	0.110^a^
Percentage of compression	31.17% ± 11.90	26.13% ± 12.60	0.166

Abbreviations: IQR, interquartile range; SD, standard deviation.

^a^ With Welch correction.

## DISCUSSION

The purposes of this study were to investigate the return of DPP and independent ambulation in paraplegic, DPP‐negative, French bulldogs following surgical decompression for treatment of TL‐IVDE. We found that 39% (14/37) of dogs had regained DPP following surgical management. Only 6% (2/37) of dogs had regained the ability to independently ambulate by the time of discharge.

At least 4 weeks post‐surgery, an additional three dogs regained DPP (17/37, 46%) and seven dogs (19%) could independently ambulate. The time of return of independent ambulation was based on limited data available for 24 dogs given the median follow‐up of 1 month post‐surgery (Table 2). With the mean time to regain the ability to walk reported to be 7.5 weeks (range, less than 1 to 36 weeks), with 30% walking between 4 and 12 weeks after surgery and 8% walking more than 12 weeks after surgery,^11^ this study was not able to accurately determine the rate of return of ambulation.

We also identified that fewer French bulldogs suffering from a lesion affecting the L4‐S3 spinal segments regained DPP. None of the other parameters investigated was associated with a return of DPP.

Other studies have used the return of independent ambulation as the primary marker of a successful outcome, with the return of independent ambulation in deep pain negative dogs within 14 days of surgical management reported to be between 26.5% and 36.4%, increasing to 42.3% after 4–6 weeks.^6^, ^7^ Those study populations consisted predominantly of Dachshunds, accounting for over 80% of the cases in one study.^6^ Our data are not directly comparable due to the differences in the duration of hospitalisation and follow‐up; furthermore, we did not control for these variables. Even so, our data highlight a potential difference in the level of short‐term recovery of French bulldogs compared to other chondrodystrophic breeds. This breed difference has been explored in one study where the presentation and outcomes of French bulldogs and Dachshunds with TL‐IVDE were compared, with only 33% (5/15) of French bulldogs regaining independent ambulation compared to 53% (96/181) of Dachshunds.^8^ While our results are not directly comparable because the time frame for regaining independent ambulation was not reported by breed in that study,^8^ their results support the premise that there might be breed‐related differences with severe TL‐IVDE‐associated myelopathy and final neurological outcome.

It is currently unknown why French bulldogs appear to have a worse clinical outcome. It has been reported that French bulldogs are more susceptible to progressive myelomalacia, with a reported prevalence up to 33%,^8^ compared with a prevalence of 9%–17.5% in other breeds.^6^ This suggests that they may be more prone to the effect of secondary injury pathways. Furthermore, French bulldogs are more prone to extensive epidural haemorrhage compared to Dachshunds, which could also explain the difference in outcome through a greater compression of the spinal cord, possible disruption of normal perfusion and further inflammatory stimulation due to red blood cell breakdown products.^14^ However, when the interaction of breed on ambulation outcome was assessed in two large cohort studies, an interaction between the two variables was not found.^12^, ^26^ The absence of a significant relationship between breed and neurological outcome could partly be explained by an absence of French bulldogs as a breed category in either study due to insufficient numbers to allow analysis.

We also observed that significantly fewer French bulldogs regained DPP with a lesion affecting the L4‐S3 spinal segments. This lesion location and poorer neurological outcome have been previously reported,^27^ showing that the combination of absent DPP and L4‐S3 lesions indicated a poor prognosis.^27^ However, another study reported that there was no effect of location on outcome in a large population of paraplegic dogs with absent DPP, although the study only compared Dachshunds and Cocker Spaniels and did not include French bulldogs.^26^ The finding of fewer French bulldogs with L4‐S3 spinal segments regaining DPP within our study is clinically concerning because L4‐S3 lesions are more common in French bulldogs when compared to Dachshunds.^8^, ^14^ The reasons why fewer French bulldogs regained DPP with L4‐S3 lesions is unknown; however, it could be speculated that this result is intrinsically related to the breed. Cocker Spaniels have been reported to have a higher incidence of L4‐S3 lesions compared to Dachshunds^28^; however, there was no reported difference in outcome between them. As mentioned above, the location of the lesion should also form part of the decision‐making process, using L4‐S3 lesions within the subset of DPP‐negative, paraplegic French bulldogs as a potentially negative prognostic indicator.

Our study did not identify an association between age, weight or duration of GA with the return of DPP. Of these factors, age has repeatedly been shown not to be associated with outcome in dogs that are paraplegic with absent DPP.^10^, ^11^, ^26^ The correlation of weight with final outcome of dogs with TL‐IVDE is unclear, with some studies reporting no effect,^11^, ^12^ while others reporting that dogs greater than 15 kg had a less favourable outcome.^27^ One reason for this disparity could relate to different studies including both chondrodystrophic and non‐chondrodystrophic dogs within their study populations; hence, when looking specifically at one breed an association with outcome was not found. Potentially, the use of other measures, such as body condition score, may be more suited to exploring the effect of body condition and neurological outcome. The absence of a link between GA duration and the return of DPP is in contrast with one study that reported an association between shorter GA duration and regaining independent ambulatory function.^29^ Interestingly, when comparing that study,^29^ with ours, the median GA times were similar, for the two outcome groups. This suggests that our study may have been underpowered to identify an effect of GA duration.

When assessing the quantitative MRI biomarkers with prognostic potential, none were associated with the return of DPP. Of the quantitative MRI biomarkers, the length of the spinal cord T2W signal increase has been the most explored imaging biomarker, although its prognostic potential remains unclear.^6^, ^21^, ^23^, ^24^, ^30^ Where such an increase in signal in T2W images has been reported to be associated with regaining independent ambulation in a predominantly Dachshund population, both its presence compared to dogs free of this alteration and a T2W/L2 ratio greater than three, were associated with an unsuccessful outcome.^24^ In contrast, within our study, a T2W signal increase within the spinal cord parenchyma was present in all the French bulldogs irrespective of their outcome, with the median T2W/L2 ratio for both groups being greater than six. This could suggest that French bulldogs with TL‐IVDE exhibit more extensive spinal cord T2W signal increase on MRI compared to other breeds, which could represent more extensive contusion‐associated changes such as necrosis, haemorrhage, oedema and inflammation.^6^, ^31^, ^32^, ^33^

For the other quantitative MRI biomarkers, previous reports have been inconclusive, with conflicting reports for the length of spinal cord swelling, the length of spinal cord compression and the severity of spinal cord compression.^10^, ^15^, ^17^, ^19^, ^21^ The absence of an association with the severity of spinal cord compression is similar to a previous study^10^; however, our data suggested that French bulldogs might have a less severe level of compression. A previous study^10^ reported that more than half of the dogs had a compression ratio greater than 50%, while in our study the mean compression ratios for both successful and unsuccessful groups were 31% and 26%, respectively. As in other studies the previous study was centred predominantly around Dachshunds.^10^ This difference in the severity of spinal cord compression for French bulldogs compared to other breeds further supports that they might be more susceptible to the effect of secondary injury pathways. This may explain why, following decompressive surgery, fewer French bulldogs regained independent ambulation compared to other breeds. It should be noted that the differences between our results and other reports could partially be explained by our study population being more homogenous, with only one breed and only severely affected dogs evaluated. Moreover, it is worth noting that making comparisons between studies will be influenced by different protocols used in the study design.

Limitations of this study include its retrospective nature and relatively small sample size. Like all retrospective studies, data quality is reliant on the clinical records. Furthermore, there was no standardised treatment protocol for each dog, with treatment options being at the attending veterinary clinician's discretion. This was further compounded by the multicentre approach of this study, introducing further heterogenicity into the study population. Another limitation is that the length of hospitalisation was not standardised, despite using the final neurological grade when the dog left the hospital (discharge or was euthanased/died) to categorise cases into groups for analysis of co‐variates. A further limitation was the challenge of obtaining long‐term follow‐up information, given the time lapse in some cases between treatment and when this study was performed, during which time dogs could have died and/or been euthanased due to their clinical condition and contacting their caregivers would be inappropriate. Follow‐up was limited for 13 of 27 cases in that the time to return of DPP was not known. The use of different imaging protocols and MRI systems of different magnet strengths by the two referral centres meant that a slightly more heterogeneous pool of images was available. The use of consensus‐based results and the short interpretation period for all the images aimed to decrease the effects of this heterogeneity as much as possible. Lastly, the dogs with a successful outcome (where data on return of DPP were available) all had a return of DPP within 8 days. The ‘unsuccessful’ group included dogs that were euthanized within 8 days, including cases 4 and 20, which were euthanased on days 5 and 4, respectively. It is possible, other factors around the decision to euthanase withstanding, that these dogs, had they lived, may have had a return of DPP.

In conclusion, our findings should be incorporated into clinical decision making and further investigations into the neurological recovery of French bulldogs following severe TL‐IVDEs are warranted.

## AUTHOR CONTRIBUTIONS

Gareth Michael Couper Jones, Alberta De Stefani, Francisco Llabres‐Diaz and Abby Caine conceived and designed the project. Gareth Michael Couper Jones and Giunio Bruto Cherubini acquired the data. Francisco Llabres‐Diaz and Abby Caine analysed the imaging data. Gareth Michael Couper Jones analysed and interpreted the data. Gareth Michael Couper Jones wrote the manuscript. All authors revised and approved the final manuscript.

## CONFLICTS OF INTEREST STATEMENT

The authors declare they have no conflicts of interest.

## ETHICS STATEMENT

The study protocol and design were approved by the Royal Veterinary College's Social Science Research Ethical Review Board (URN SR2020‐0234). The data collected in this trial are collated and stored at the Royal Veterinary College, London.

## Supporting information

Supporting Information

## Data Availability

The raw data described in this article is available from the corresponding author, upon reasonable request.
