# Supplementary material for: A case series of 37 surgically managed, paraplegic, deep pain negative French bulldogs, with thoracolumbar intervertebral disc extrusion, from two English referral centres
Source: Vet Rec Open. 2023 May 9;10(1):e61. doi: 10.1002/vro2.61 (PMC10170243; doi:10.1002/vro2.61)
Supplement: Supplementary file 1 — Supporting Information [file VRO2-10-e61-s001.pdf]

Supporting Information

Quantitative MRI imaging methodology

The following criteria were evaluated for each case (details in subsequent paragraphs) and a consensus reached for each one

- Length T2 signal increase/L2 length ratio
- Length compressive extradural lesion/L2 length ratio
- Severity of the spinal cord compression - (%).
- Length of the spinal cord swelling (lack of Cerebrospinal fluid (CSF) signal or cord swelling from traditional sequences)/L2 length ratio.

All lengths were measured in centimetres (cm).

Length of T2W signal increase/L2 length ratio

The length of T2W signal increase of the spinal cord was defined as the length of spinal cord parenchyma where the alterations in T2W signal were taller or wider than what further cranially or caudally could be seen as central canal dilation (or syringomyelia if present, Figure S1). This differentiation was aided by evaluation of transverse images when available. The height or width, depending on the evaluated plane, of the signal increase could be slightly uneven or intermittent, particularly immediately dorsal and in the immediate vicinity of the extradural compressive lesion, but this did not preclude its inclusion within the measured length of abnormal spinal cord. (If the most compressive lesion had led to a focal loss of T2 signal in-between altered areas, not uncommon at the point of maximum cord compression, this diseased area was still included).

In dogs where the vertebral column demonstrated marked curvature on the sagittal plane, the length of the abnormality was measured adding the length of several straight lines to represent as accurately as possible the true length of affected spinal cord. Furthermore, if a thoracolumbar transitional vertebra was present, L2 was identified counting from L7 cranially.

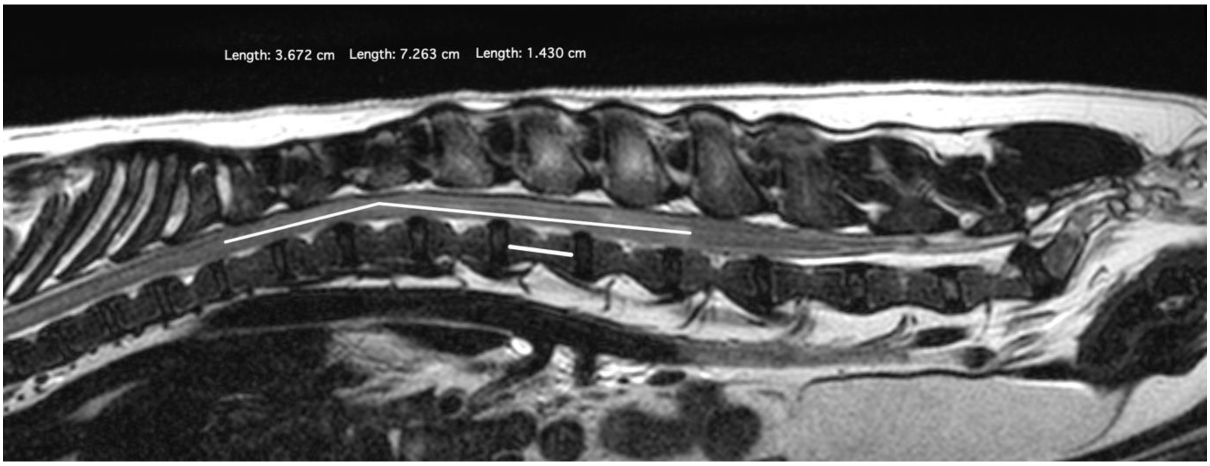

Figure S1 - Sagittal T2W image of the vertebral column and spinal cord of a 5-year, 1-month-old, male entire, French bulldog demonstrating how straight measurements of the length of the T2 signal change were obtained in this case and then added to obtain one single T2 length measurement per case.

Length of compressive extradural lesion/L2 length ratio
Similar to the above, the total length of the lesion/s causing spinal cord compression was calculated, even if their width / volume varied within their length (Figure S2). Any indentation of the spinal cord was taken as a sign of compression, even if mild. Again, added lengths were used if the spinal cord followed the natural curvature of the vertebral column. Discrimination of extruded disc material from other extradural components of the compression – haemorrhage, for instance – was not attempted. All available planes and sequences could be used to determine the length of the compressive lesion (Figure S3), with transverse plane images always evaluated when available, but the measurement was obtained in the sagittal plane.

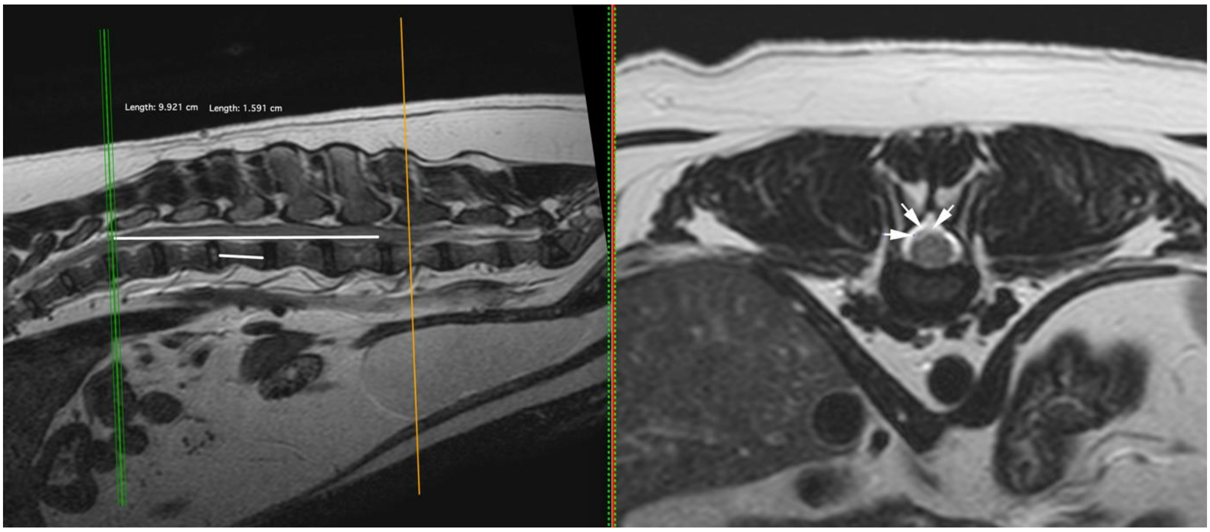

Figure S2 - Screen capture from Horos of a 4 year 6 month old, male neutered, French bulldog demonstrating the length of compressive lesion measured on this dog on the sagittal plane using transverse images, when available, to detect the presence of compressive lesions at different levels.

Left panel-midline sagittal T2W image.

Right panel - transverse T2W image at the level of the green line on the sagittal image, demonstrating the presence of a mildly compressive dorsal extradural lesion (arrows). L2 length also measured.

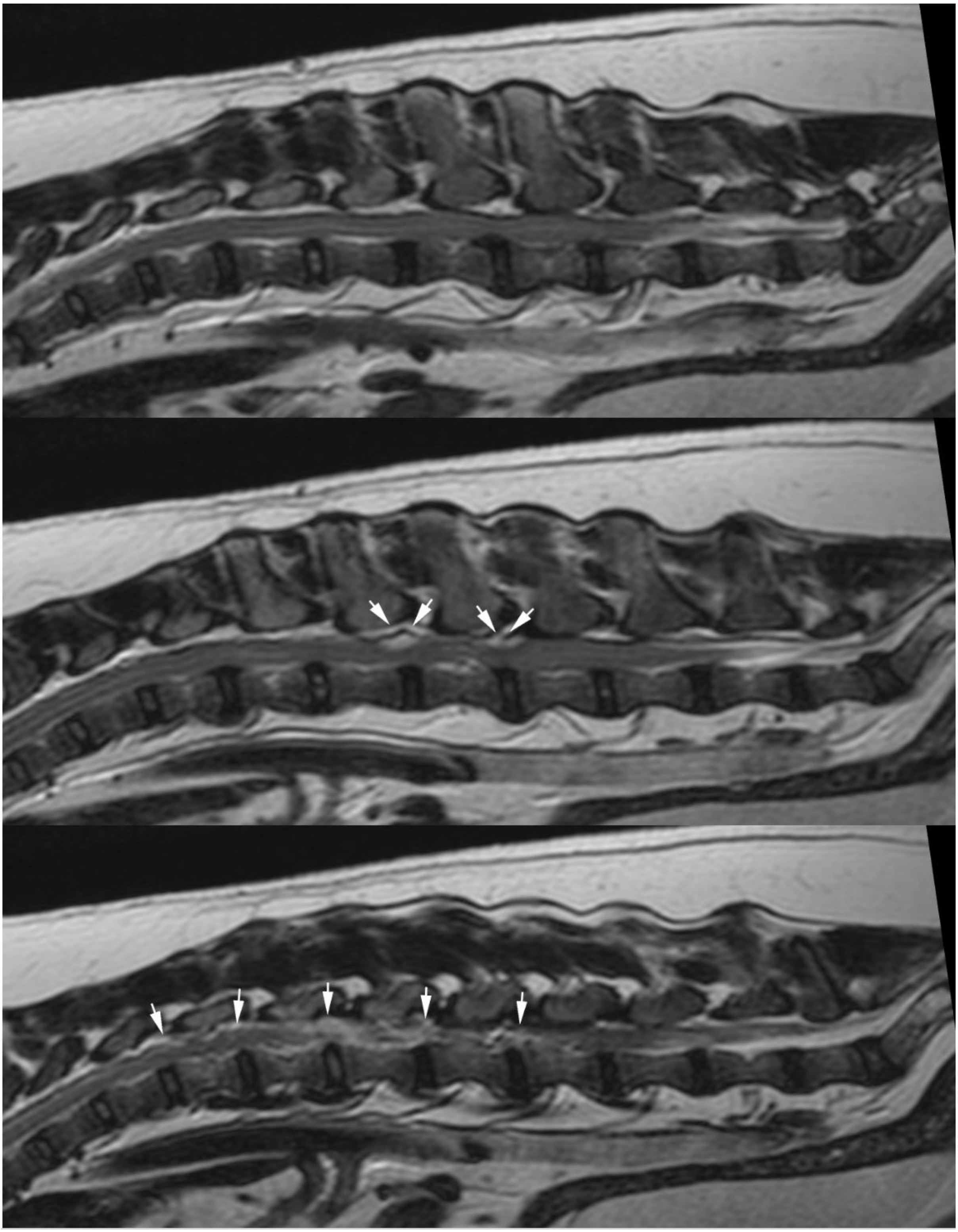

Figure S3 - Consecutive sagittal T2W images, from midline to the left, same dog as figure S2, demonstrating the use of parasagittal images to detect the presence of disperse extradural lesions of different volumes (mid image, arrows, most compressive material; bottom image, arrows, less compressive but more diffuse lesion).

Length of spinal cord sweeling /L2 length ratio

The length of spinal cord swelling was defined as the length of spinal cord demonstrating total loss of normal CSF signal on sagittal T2 and particularly STIR sequences, compared to the length of L2 (Figure S4). Transverse plane images were always evaluated to confirm the lack of CSF signal, when available.

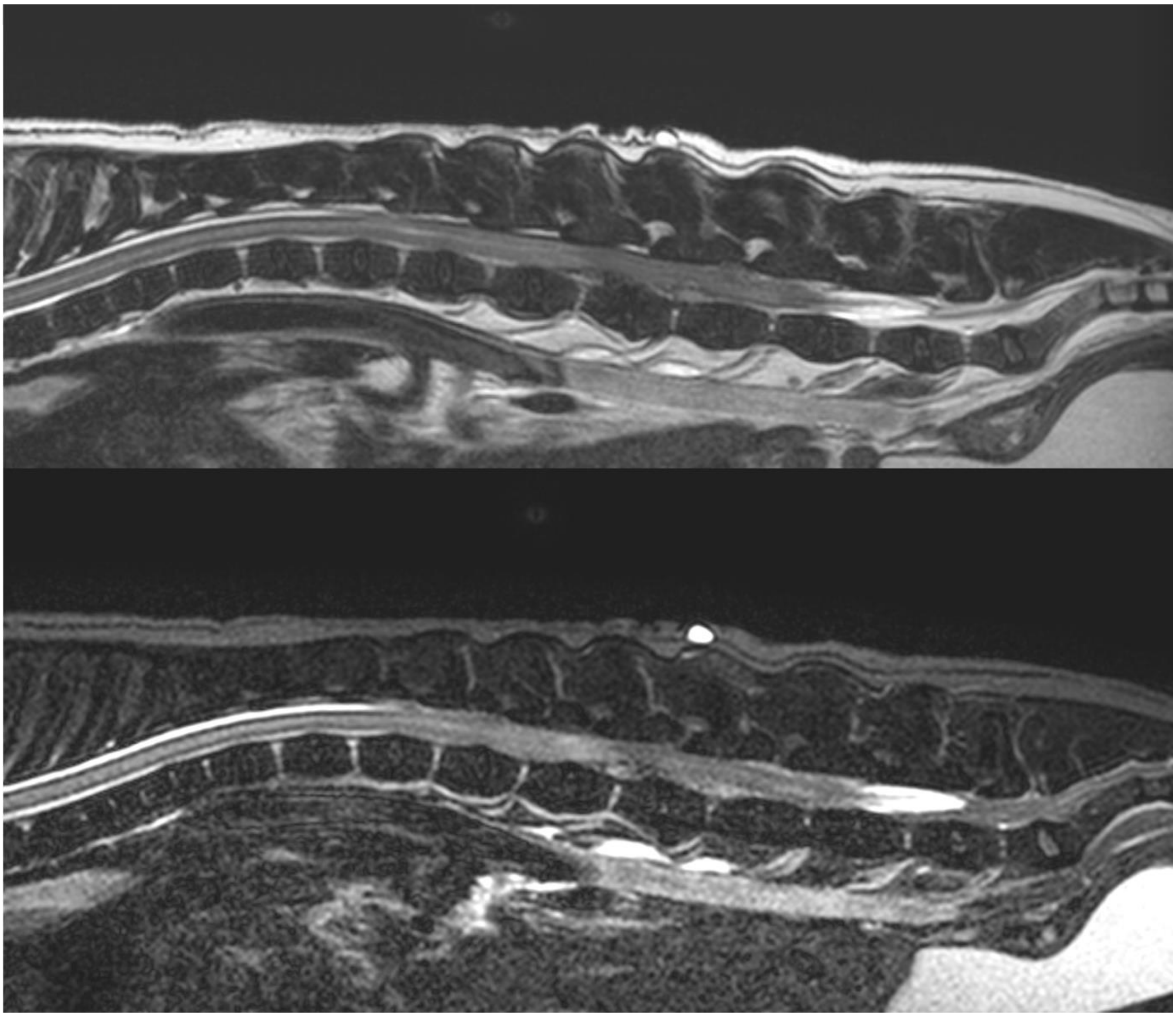

Figure S4 - Midline sagittal T2W (top image) and STIR (bottom image) of a 4-year, 10-month-old, female neutered, French bulldog. Lack of CSF, interpreted as an indirect sign of cord swelling, is better assessed on the STIR image.

Severity (percentage) of compression

The severity of compression was defined by calculating the height or width (depending on the main direction of the compression) at the point of maximum compression (C) and comparing this to the height or width, respectively, of the normal (N) spinal cord at the centre of the vertebral body cranial to the last compressive lesion (Figure S5). If the curvature of the cord meant that a true height could not be measured on the transverse plane, the sagittal view was used instead. If the width was used for C then N was measured in the same direction. This compression was expressed as a percentage (N-C)/N*100.

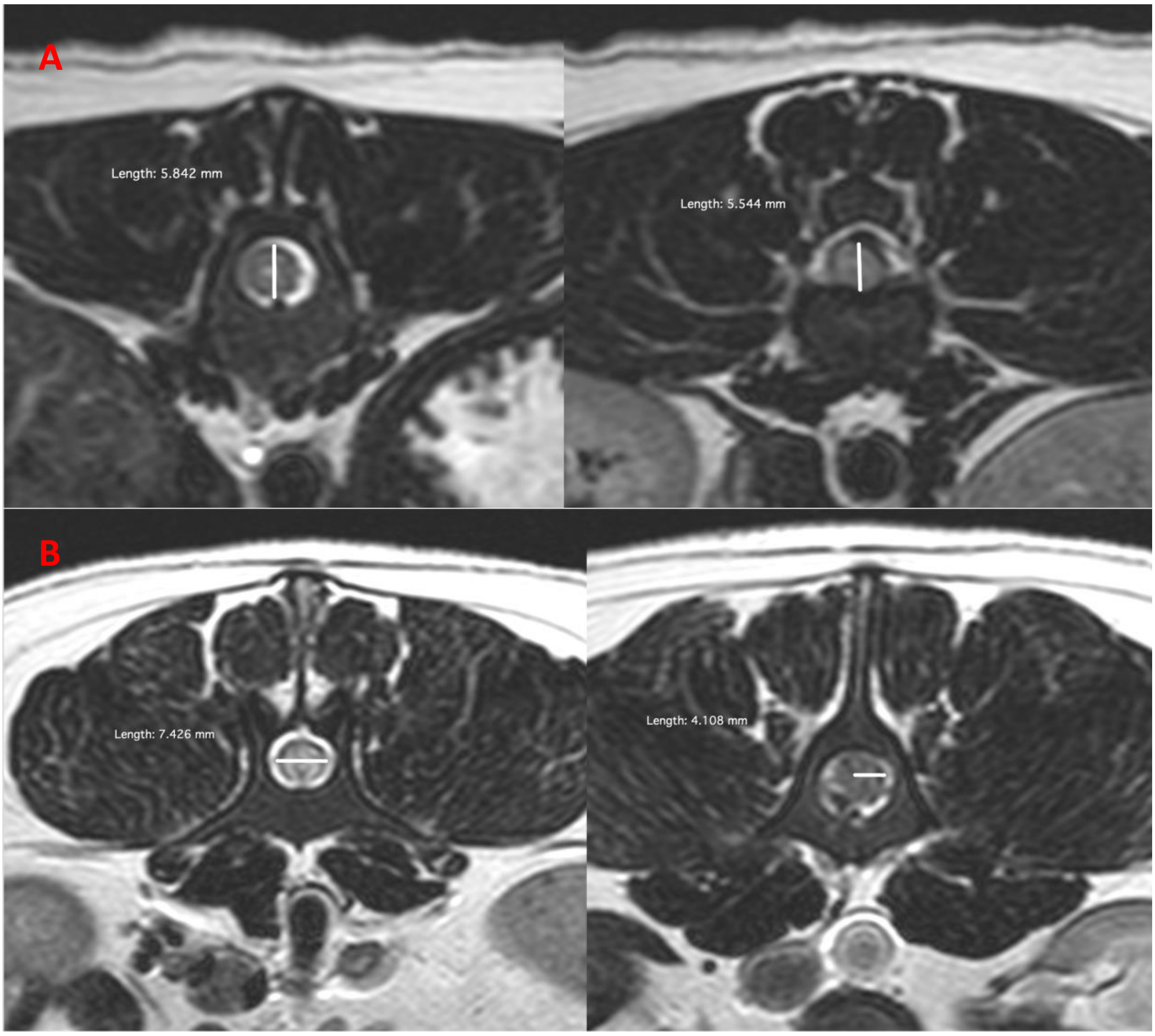

Figure S5

(A) Transverse T2W images of a three-year, 6-month-old, male neutered, French bulldog demonstrating the methodology behind the calculation of the severity of compression, using cord height in this case.

(B) Transverse T2W images of a 4-year, 11-month-old, male neutered, French bulldog demonstrating the use of cord width to evaluate the degree of cord compression.
